# Protein Hydrolysate from Waste of Catfish Fillet Processing for Snakehead Fish Feed Formulation

**DOI:** 10.1155/2023/2815122

**Published:** 2023-12-29

**Authors:** Mulyasari, Subaryono, Bagus Sediadi Bandol Utomo, Yosmaniar, Imam Taufik, Muhamad Yamin, Irin Iriana Kusmini, Tri Marwati

**Affiliations:** ^1^Research Center for Marine and Land Bioindustry, National Research and Innovation Agency, North Lombok, West Nusa Tenggara 83352, Indonesia; ^2^Research Center for Fisheries, National Research and Innovation Agency, Bogor, West Java 16911, Indonesia; ^3^Research Center for Applied Zoology, National Research and Innovation Agency, Bogor, West Java 16911, Indonesia; ^4^Research Center for Food Technology and Process, National Research and Innovation Agency, Gunung Kidul, Yogyakarta 55861, Indonesia

## Abstract

The negative impact of fish processing industry waste needs to be minimized, by processing it into valuable products, one of which is fish feed. The objectives of this research were to determine the optimum dose of crude extract of *B. cereus* RGL.1.1 enzyme in hydrolyzing protein from the waste of catfish (*Pangasius hypophthalmus*) fillet processing and to evaluate the effectiveness of using protein hydrolysate in snakehead fish (*Channa striata*) feed. There were four doses of enzyme treatment for protein hydrolysis designed in a completely randomized design, namely, 0, 4, 6, and 8% (v/w) with three repetitions. Furthermore, to assess the quality of protein hydrolysate, an analysis of soluble protein level, hydrolysis degree, amino acid content, fatty acid content, and digestibility was carried out. The percentage of protein hydrolysate applied in the feed formula was 0, 15, 30, and 45% (v/w), which was designed in a completely randomized design with three replications. Absolute weight growth, specific growth rate, protein efficiency ratio, feed efficiency, and snakehead fish survival were measured to evaluate the effects of the feed formula. Results showed that the crude extract of *B. cereus* RGL.1.1 enzymes at a concentration of 6% (v/w) enhanced the availability of soluble proteins, amino acids, fatty acids, and feed digestibility. Protein hydrolysate application in snakehead fish feed formula up to 45% (v/w) can improve the growth performance (8.03%), protein efficiency ratio (25.66%), and feed efficiency (23.41%).

## 1. Introduction

Waste in the fish processing industry can become a fundamental problem for our environment; it must be managed and treated seriously to reduce its negative impacts. Fish waste, if treated and processed properly, can not only reduce the environmental problem but also be beneficial to our lives. One of the beneficial products of fish waste is fish feed. The fish waste can be used as a source of raw materials for fish feed because the waste consists of inedible parts of fish such as offal, skin, and bones that are still rich in protein and minerals and can be used as feed ingredients, especially after being processed into protein hydrolysates. This type of protein can replace fish meals as a protein source [1]. This hydrolysate is frequently used as a protein supplement and appetite stimulant [1, 2] and has been shown to boost fish growth and feed efficiency [2–4].

Protein hydrolysate can be produced using protease enzymes. Enzymes can help reduce the risks of product deterioration and damage [5]. Protease (E.C.3.4.21) is an enzyme of the hydrolase group that helps in breaking down peptide bonds in protein molecules. This enzyme catalyzes the hydrolysis process involving the addition of water to a specific substrate bond [6]. The protease enzyme is found in many organisms, including plants, animals, and microbes. Microorganisms are regarded to offer advantages when utilized as enzyme producers since they are quick to generate, easy to grow, and whose environmental circumstances can be adjusted [7]. One type of microorganism that can produce the protease enzyme is bacteria, one of which is *Bacillus cereus* [8–10]. *B. cereus* RGL1.1, isolated from hot springs in the Rengganis crater, Bandung Regency [11], can be used to produce proteases that can hydrolyze fish protein hydrolysates. However, the optimum dosage of protease from *B. cereus* RGL1.1 for catfish protein hydrolysis is still unknown. As a result, more research is needed to determine the optimum dose of the enzyme to produce protein hydrolysates and to evaluate the impact of these hydrolysates on snakehead fish growth and digestibility.

The natural population of snakehead fish has declined because of overfishing. Aquaculture is the best alternative to ensure snakehead fish's long-term production. Nonetheless, there are problems with its implementation, particularly in the availability of feed. Since fish meals and soybean meals must be imported from other countries, their prices are high in Indonesia. Furthermore, predatory fish, such as snakehead fish, require high protein levels in their feed to grow properly. Snakehead fish grow best when fed with a 40% protein diet [12]. For these reasons, it is necessary to find an alternative feedstuff of low price, one that is readily available and contains sufficient nutrients. The best option so far is to use the waste of the catfish fillet industry, which is prevalent in Indonesia.

The objective of this experiment was to maximally use the waste of catfish (*Pangasius hypophthalmus*) fillet processing industry to produce feed for snakehead fish (*Channa striata*) by establishing the optimum dose of crude protease to produce protein hydrolysate, snakehead fish feed formulation, and to observe the effects of protein hydrolysates on the feed digestibility and the growth rate of the fish.

## 2. Materials and Methods

### 2.1. Materials

Fish waste was obtained from CV Kurnia Mitra Makmur Purwakarta, West Java, a catfish (*Pangasius hypophthalmus*) fillet processing company. The waste was taken in the frozen condition; it was practically fish offal including the intestine which was cleaned from fish excrement before being frozen.

Other materials used in this research were *B. cereus* RGL1.1 (isolated from hot springs in the Rengganis crater, Bandung Regency, Indonesia), aquadest (CV. Setia Guna, Indonesia), nitrogen and hydrogen gases (PGN, Indonesia), trypticase soy broth, trypticase soy agar, trichloroacetic acid, Bradford reagent, bovine serum albumin, chromium, sodium hydroxide, sulfuric acid, boric acid, chloride acid, hydrogen peroxide, petroleum benzene, nitric acid, perchloric acid, o-phthalaldehyde, methanol, sodium acetate, ethanol, n-hexane, boron trifluoride, sodium sulfate, and sodium chloride (Merck, Germany).

### 2.2. Determination of the Dosage of Fermentation Broth

To hydrolyze the catfish offal, the experiment employed a fermentation broth of *B. cereus* RGL1.1. The bacteria were cultivated in tryptic soy broth media under static conditions at a density of 10^8^-10^9^ CFU/mL for 48 hours at 28°C with intermittent shaking to obtain the fermentation broth. After centrifuging the fermentation broth at 10,000 rpm for 30 minutes, the supernatant was collected. The procedure for producing the protein hydrolysate was based on [13]. Before hydrolysis, fish offal is defrosted and chopped until crushed and then homogenized with aquadest in a ratio of 1 : 3. The mixture is then adjusted to *pH* 8. The catfish offal was hydrolyzed for 6 hours at 55°C using the fermentation broth at concentrations of 0%, 4%, 6%, and 8% (v/w). The hydrolysate is heated to 80°C for 20 minutes to deactivate enzymes. Afterward, the sample is centrifuged for 20 minutes at 5000 x g to separate the supernatant from the sediment. The protein hydrolysates in the form of supernatants are then analyzed further. In order to evaluate the quality of the protein hydrolysate, the Bradford method was employed to measure the soluble protein content. This method determines the total protein concentration present in a sample by measuring the binding of protein molecules to Coomassie dye under acidic conditions, which changes color from brown to blue. Additionally, the quality of the protein hydrolysate was further assessed by measuring the protein hydrolysis degree using the method suggested in [14]. This method makes use of a nitrogen soluble index to determine the degree of hydrolysis by using trichloroacetic acid as a precipitating agent.

After producing the protein hydrolysate at the optimal dose, further analysis was conducted to determine its amino acid and fatty acid profiles, as well as its digestibility. The optimal dose is the one that produces the highest protein hydrolysates. To analyze the amino acid profile, high-performance liquid chromatography (HPLC) was used with a C-18 column, a flow velocity of 1 mL/min, and a pressure of 3000 psi. The fluorescence detector with a wavelength of 350–450 nm was employed, and the device temperature was set to 27°C. The mobile phase consisted of 95% methanol and 1 M sodium acetate. For the fatty acid profile, gas chromatography (GC) was used. The standard used for the analysis was SupelcoTM 37 Component FAME Mix. Nitrogen gas was used as the mobile phase, with a pressurized flow rate of 20 mL/min, and hydrogen gas served as the burner gas with a flow rate of 30 mL/min. The analysis was performed using a 60-meter-long Quadrex Fused Silica Capillary Column 007 Cyanoprophyl Methyl Sil, with an inner diameter of 0.25 mm. The temperature was maintained at 125°C and was gradually increased by 5°C per minute until it reached 225°C. The injector temperature was 220°C, and the detector temperature was 240°C. In order to test the digestibility of the protein hydrolysate, the method suggested in [15] was used with 0.5% Cr_2_O_3_ as an indicator. For the tests, a feed mixture was prepared using 70% commercial feed and 30% fish protein hydrolysate, and the optimal dosage was used as a reference. Commercial feed was used as a control. The experiment was conducted in a 60 × 50 × 40 cm aquarium filled with 100 L of water and equipped with an aeration system. The feeding experiment was carried out using twenty snakehead fish weighing 10 g each, with a density of twenty individuals per aquarium. Satiated feeding was done three times a day. After five days of feeding, feces were collected by siphoning and sieving them through a fine sieve. To avoid nutrient decomposition in the water, feces were collected an hour after feeding. The feces were then dried in a 40°C oven for 24 hours, wrapped in sealed plastic bags, and stored in a refrigerator at 5–6°C. The process of collecting fecal samples was conducted until enough samples were available for analysis. Both the feed and fecal samples were analyzed for their proximate and chromium (Cr_2_O_3_) content. For the proximate analysis, crude protein was measured using the Auto Kjeldahl System (VELP Scientifica, Milano, Italy), crude lipid content was measured through the ether-extraction method using a Soxhlet extractor (Foss - ASN 3125-2005), moisture content was measured by drying the samples in an oven at 105°C for 4 hours, and the ash content was measured using a furnace (550 ± 20°C for 3 hours). In chromium analysis, approximately, 0.2 grams of ground samples were digested with the oxidation process, and the final solution was measured through simple photometry at 350 nm.

### 2.3. The Effectiveness Test of the Use of Protein Hydrolysate in Snakehead Fish Feed

A completely randomized design with four treatments and three replications was used to determine the effectiveness of using protein hydrolysate in snakehead fish feed. The four treatments of the percentage of protein hydrolysate applied in the feed formula were 0, 15, 30, and 45%. The commercial feed used was “Prima Feed 1000” (*PF*1000) produced by CV Matahari Sakti. The protein hydrolysate was mixed thoroughly with the mashed commercial feed before being transformed into pellets. The proximate composition of the feed after the addition of protein hydrolysate is presented in Table 1.

Snakehead fishes (*C. striata*) weighing 38.19 ± 0.14 g were acclimated for 14 days before being stocked in aquariums at a density of twenty individuals per aquarium. Fish are collected before and after treatment for proximate analysis. The experiment lasted 40 days. Feeding treatment was administered three times a day. The test parameters were absolute weight growth (AWG) [16], specific growth rate (SGR) [17], feed efficiency (FE) [15], protein efficiency ratio (PER) [18], and survival rate (SR) [16]. The formula for each parameter is as follows:

- Absolute weight gain (AWG) = Final weight (g) − Initial weight (g)
- Feed efficiency (FE) = (Fish wet weight gain/feed intake) × 100
- Specific growth rate (SGR) = ((ln final weight − ln initial weight)/feeding period in days) × 100
- Protein efficiency ratio (PER) = Fish wet weight gain/protein intake
- Survival rate (SR) = (Final number of the remaining fish/initial number of fish) × 100

### 2.4. Data Analysis

The software program SPSS verse 22.00 was used to perform statistical tests. Data of the test parameters were collected and analyzed using analysis of variance (ANOVA); if the results were different significantly, the Duncan test at a 95% confidence interval was applied.

## 3. Results and Discussion

### 3.1. Determination of Enzyme Dosages for the Protein Hydrolysis Process

**Figure 1:**
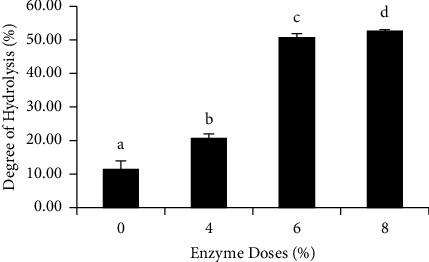
The degree of hydrolysis of the catfish offal, which was hydrolyzed using a fermentation broth of *B. cereus* RGL1.1. Data were expressed as mean ± SD. Different superscript letters in the bars have significantly different results (*P* < 0.05).

The degree of hydrolysis was significantly different in each concentration of *B. cereus* RGL1.1 fermentation broth (*P* < 0.05) (Figure 1). The highest degree of hydrolysis of catfish offal was observed with a fermentation broth concentration of 8%, followed by concentrations of 6% and 4%, respectively. The control had the lowest degree of hydrolysis. This finding was in line with the findings of [19], where the authors used the enzyme papain to hydrolyze snakehead fish fillet waste and discovered that the higher the concentration of papain, the greater the degree of hydrolysis produced. According to [20], the higher the enzyme concentration, the higher the level of dissolved nitrogen in protein hydrolysates due to peptide bond breakage.

**Figure 2:**
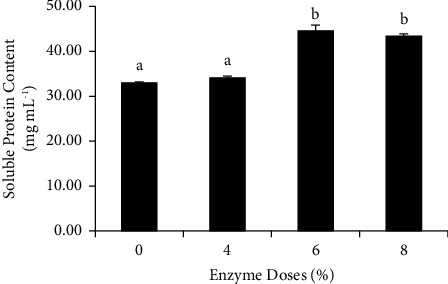
Soluble protein (mg/mL^−1^) protein hydrolysate from the catfish fillet processing waste (Data were expressed as mean ± SD). Different superscript letters in the bars show significantly different results (*P* < 0.05).

The substantial degree of hydrolysis in the 6% and 8% concentrations is further verified by the soluble protein analysis (Figure 2). The higher the concentration of the fermentation broth, the higher the level of soluble proteins. A rise in the concentration of soluble proteins implies that protein bonds are broken down into peptides or amino acids. Insoluble proteins are transformed into soluble nitrogen compounds during the hydrolysis process, which are subsequently broken into simpler compounds, such as peptides and amino acids, which are more easily absorbed by the body [19]. Although a concentration of 8% results in the highest increase in hydrolysis and soluble protein, it is statistically insignificantly different from a concentration of 6% (*P* > 0.05). As a result, protein hydrolysate at a dose of 6% was chosen for the next research step.

Except for lysine, practically, all the amino acids tested in catfish offal hydrolysate have increased (Table 2). Transamination occurs throughout the hydrolysis process, increasing the number of amino acids liberated [21]. The dominant amino acids in catfish hydrolysate in this study were glutamic acid, lysine, aspartic acid, leucine, and isoleucine. This is slightly different from turbot fish protein hydrolysate where the dominant amino acids are glycine, glutamic acid, aspartic acid, and alanine [22].

The fatty acid composition of catfish viscera hydrolyzed with *B. cereus* RGL1.1 is shown in Table 3. Apart from proteolysis, the *B. cereus* RGL1.1 crude enzyme extract produces lipolysis in catfish offal, resulting in alterations in its fatty acid composition. It is suspected that besides protease, the crude extract of enzymes extracted from the bacteria *B. cereus* RGL.1.1 contains lipase, which can break down fats into fatty acids. This is evident from the increase in the quantity of almost all types of fatty acids measured, such as myristate, stearic, oleic, linoleic, linolenic, EPA, and DHA. According to Subandiyono and Hastuti [23], freshwater fish require essential fatty acids such as linoleic acid and/or linolenic acid for normal growth and development. The results of this study suggest that catfish waste protein hydrolysate can be a potential source of essential fatty acids that can be used in freshwater fish feed.

Catfish offal protein hydrolysate digestibility differs significantly (*P* < 0.05) from that of the control (Table 4). The use of a fermentation broth from *B. cereus* RGL.1.1 resulted in higher protein digestibility than the control in this research. This study's findings support the claim by [24] that high protein digestibility in fish protein hydrolysates is associated with increased solubility and the release of protein molecular structure bonds into smaller peptide units during hydrolysis. The benefits of utilizing protein hydrolysate in digestibility, according to [25], are related to the composition of the resultant product, which comprises peptides with a low molecular weight that contribute to enhanced digestibility.

Fat digestibility rose significantly (*P* < 0.05) in this study when compared to controls. Fat digestibility increased from 80.10 ± 0.68% to 85.61 ± 1.45%. The increase in fat digestibility found in this study is assumed to be related to lipase enzyme activity in *B. cereus* RGL1.1. *B. cereus* exhibits lipase activity, and it can degrade fats into simple molecules [26, 27].

### 3.2. The Efficacy of Using Catfish Offal Protein Hydrolysate in Snakehead Fish Feed

The effectiveness of applying protein hydrolysate from catfish offal for snakehead fish feed has been studied and it was discovered that adding up to 45% of the protein hydrolysate tends to further enhance the fish's growth. This is supported by the increase in snakehead fish's absolute weight growth (AWG) and specific growth rate (SGR) (Table 5).

The result of this study agrees with the findings of the authors of [28], who found that protein hydrolysate could increase the growth of the *Pseudosciaena crocea* R fish. The authors of [29] found that using 30% fish protein hydrolysate resulted in a higher tilapia growth performance. Although the growth trend of snakehead fish fed with catfish offal protein hydrolysate in this study has increased, the DGR and AWG of control (dose of 0%) were not statistically different (*P* > 0.05)with dosages of 15% and 30%. However, the controls (0%) differed significantly (*P* > 0.05) with a dosage of 45%. This demonstrates that up to 45% dose of protein hydrolysate can increase snakehead fish growth. According to reference [30], fish protein hydrolysate has been shown to increase survival and growth rate, lower malformation levels, increase enzyme activity, modify nutrient transformation patterns in the digestive tract, increase nutrient absorption, and induce the formation of nonspecific immune responses in larvae, fry, and adult fish at certain levels. The authors of [31] stated that a moderate inclusion of fish protein hydrolysate in aquafeeds has the potential to improve growth, feed utilization, immune functions, and disease resistance of fish.

The feed efficiency (FE) at doses of 15% and 30% did not differ significantly (*P* > 0.05) from that of the control (concentration of 0%), though it tended to have a higher trend than the control. Whereas, FE at 45% is higher and significantly different from FE at 0% (*P* < 0.05) (Table 5). The FE of snakehead fish-fed protein hydrolysate in this study was higher (37.42–46.18%) than the FE of snakehead fish fed gold snail meal (12.74–21.47%). The authors of [32] reported that the feed efficiency of snakehead fish decreased after being fed with golden snail meal. On the contrary, when snakehead fish are fed with hydrolysate of catfish offal, feed efficiency is increased. Fish protein hydrolysate is considered a suitable source of protein for human and animal nutrition due to its balanced amino acid composition and low molecular weight, allowing for a higher rate of absorption by the intestines [30]. There is a suspicion that the low PER (protein efficiency ratio) value observed in the 30% dose treatment is due to genetic variation among the fish used in the experiment. Despite the fact that at the start of the experiment, all fish were obtained from the same parent source and were of similar size (as per the complete randomized design), there is still a possibility of genetic variations that influence the growth rate during the experiment.

Statistically, the protein efficiency ratio (PER) values of snakehead fish-fed hydrolysate protein at 15% and 45% doses differed significantly (*P* < 0.05) from those at a dose of 30% and control (without hydrolysate protein) (Table 5). It is suspected that the high value of protein efficiency ratio observed at the doses of 15% and 45% is due to the presence of protein hydrolysate of catfish in the feed. This hydrolysate has been converted into simple amino acids and peptide compounds, which are easily digestible by the snakehead fish and therefore, can be utilized optimally for its growth. The PER parameter is widely considered a good criterion for assessing the protein quality in aquatic feed. The difference in PER values indicates a difference in feed protein quality [33].

When snakehead fish consume protein hydrolysates, their protein content rises from 15.60% to around 16.28-17.24% and their fat content rises from 2.93% to around 3.51–4.00%. Meanwhile, the ash content has dropped from 5.13% to between 4.80 and 5.04% (Table 6). The high protein level in snakehead fish treated with hydrolyzed offal catfish compared to controls indicates that up to 45% of such material is sufficient to deposit the protein in the body (Table 6). This is consistent with the findings of the authors of [34], who found that up to 50% fish protein hydrolysate in carp juvenile feed is sufficient to induce muscle protein deposition. The same thing can be seen in snakehead fish body fat content, where the addition of catfish offal hydrolysate in snakehead fish feed is significantly higher (*P* < 0.05) than in the control. According to several studies, fish protein hydrolysate raises fat levels in fish [35–38].

**Figure 3:**
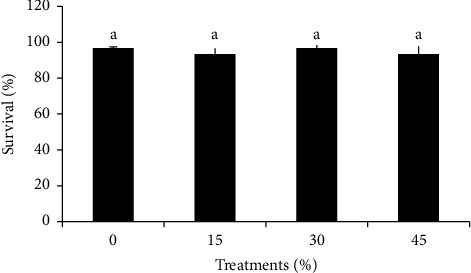
The survival rate (%) of snakehead fish fed with feed containing protein hydrolysate from catfish offal (Data were expressed as mean ± SD). The same superscript letters in the bars show no significantly different results (*P* > 0.05).

The various analyses demonstrated that adding hydrolyzed catfish offal to the meal had no significant effect on snakehead fish survival (*P* > 0.05) (Figure 3). The survival rate of snakehead fish was relatively high during the study, ranging from 93.33 to 96.67%. This implies that adding 45% catfish offal protein hydrolysate to snakehead fish feed is still relatively safe and does not affect the fish's survival. This research showed that the survival of snakehead fish after being fed hydrolysate protein was in line with other studies such as [24, 33]. Fish protein hydrolysates, according to [30], can promote fish survival and growth. The authors of [25] reported the same thing, that fish protein hydrolysate in diet can improve tilapia larvae' survival by stimulating the animal's immune system. The authors of [39] also said that the presence of fish hydrolysates may also improve the digestion, absorption, and utilization processes of animals and increase their immunity.

## 4. Conclusions

The hydrolysis of catfish fillet processing byproducts using crude extract enzyme *B. cereus* RGL.1.1 improves the availability of soluble proteins, amino acids, and fatty acids as well as the digestibility of this feedstuff. In the process of producing protein hydrolysates from catfish fillet processing waste, a 6% enzyme dose can currently be used. Meanwhile, utilizing up to 45% catfish offal protein hydrolysate in feed can improve snakehead fish growth performance, protein efficiency ratio, and feed efficiency.

## Figures and Tables

**Table 1:** The proximate composition of snakehead fish feed after the addition of different doses of protein hydrolysate.

Protein hydrolysate dose (%)	Proximate (%)				
	Crude protein	Crude fat	Ash	Crude fiber	Nitrogen-free extract
0	39.68	5.52	10.61	1.90	42.31
15	39.81	5.22	11.07	1.61	42.30
30	39.73	5.46	10.95	1.99	41.98
45	40.04	5.65	11.8	1.85	41.15

**Table 2:** Amino acid composition of protein hydrolysate from catfish fillet processing waste with and without hydrolysis.

No.	Type of amino acid	Amino acid composition (%)	
		With hydrolysis	Without hydrolysis
1	Aspartic acid	1.567	1.487
2	Glutamic acid	3.001	2.679
3	Serine	0.730	0.578
4	Glycine	0.725	0.656
5	Histidine	0.556	0.496
6	Arginine	0.409	0.388
7	Threonine	0.764	0.574
8	Alanine	0.620	0.586
9	Proline	0.590	0.548
10	Tyrosine	0.663	0.592
11	Valine	0.753	0.714
12	Methionine	0.569	0.526
13	Cysteine	0.440	0.368
14	Isoleucine	0.851	0.755
15	Leucine	0.920	0.759
16	Phenylalanine	0.699	0.597
17	Lysine	1.721	1.806

**Table 3:** Fatty acid composition of protein hydrolysate from catfish fillet processing waste with and without hydrolysis.

No.	Type of fatty acid	Fatty acid composition (%)	
		With hydrolysis	Without hydrolysis
1	Laurate	0.027	0.032
2	Myristate	0.464	0.335
3	Palmitate	2.452	2.658
4	Stearic	7.652	7.072
5	Oleic	32.715	31.584
6	Linoleic	10.565	10.267
7	Linolenic	0.386	0.362
8	Docosahexanoic acid (DHA)	3.652	3.339
9	Eicosapentanoic acid (EPA)	1.528	1.374

**Table 4:** Snakehead fish (*C. striata*) digestibility after being fed feed that contains protein hydrolysate from catfish fillet processing waste.

Treatments	Digestibility value (%)	
	Protein	Fat
Control feed	85.85 ± 0.19^a^	80.10 ± 0.68^a^
Feed with protein hydrolysate	89.97 ± 0.99^b^	85.61 ± 1.45^b^

Data were expressed as mean ± SD. Different superscript letters in the same column show significantly different results (*P* < 0.05).

**Table 5:** AWG, SGRFE, and PER snakehead fish-fed hydrolysate protein from catfish waste in concentrations of 0, 15, 30, and 45%.

Parameter	Protein hydrolysate dose			
	0%	15%	30%	45%
AWG (g)	3.49 ± 0.18^ab^	3.48 ± 0.16^ab^	2.58 ± 0.34^a^	3.77 ± 0.06^b^
SGR (%/day)	1.62 ± 0.06^a^	1.62 ± 0.06^a^	1.60 ± 0.01^a^	1.71 ± 0.02^a^
FE (%)	37.42 ± 5.00^a^	45.40 ± 2.39^ab^	44.35 ± 2.96^ab^	46.18 ± 1.67^b^
PER (%)	1.13 ± 0.11^a^	1.49 ± 0.07^b^	1.02 ± 0.15^a^	1.42 ± 0.03^b^

Data were expressed as mean ± SD. Different superscript letters in the same line show significantly different results (*P* < 0.05). AWG (absolute weight growth), SGR (specific growth rate), FE (feed efficiency), and PER(protein efficiency ratio).

**Table 6:** Body composition of snakehead fish fed protein hydrolysate-containing feed at dosages of 0, 15, 30, and 45%.

Protein hydrolysate dose (%)	Body composition (%)			
	Water	Protein	Fat	Ash
0	73.52 ± 0.31	15.60 ± 0.26^a^	2.93 ± 0.12^a^	5.13 ± 0.18^a^
15	72.95 ± 0.11	16.28 ± 0.12^a^	3.51 ± 0.14^b^	4.92 ± 0.10^b^
30	71.97 ± 0.61	17.24 ± 0.34^b^	4.00 ± 0.14^c^	5.04 ± 0.27^b^
45	72.92 ± 0.73	16.35 ± 0.57^ab^	3.68 ± 0.11^b^	4.80 ± 0.11^b^

Data were expressed as mean ± SD. Different superscript letters in the same column show significantly different results (*P* < 0.05).

## Data Availability

The data used to support the findings of this study are available from the corresponding author upon request.
